# Mechanical Properties and Durability Performance of Low Liquid Limit Soil Stabilized by Industrial Solid Waste

**DOI:** 10.3390/ma18020469

**Published:** 2025-01-20

**Authors:** Xiaoli Wang, Xiancong Wang, Pingfeng Fu, Jinjin Shi

**Affiliations:** 1 School of Civil and Resources Engineering, University of Science and Technology Beijing, Beijing 100083, China; xiaoliwang@ustb.edu.cn (X.W.); runner17@163.com (X.W.); 2 State Key Laboratory of Mineral Processing, Beijing 100160, China; 3 Cangzhou Municipal Engineering Company Limited, Cangzhou 061000, China; czszjsk@126.com

**Keywords:** industrial solid waste, liquid limit soil, mechanical properties, durability performance, freeze–thaw cycles, dry–wet cycles

## Abstract

To improve the mechanical and durability properties of low liquid limit soil, an eco-friendly, all-solid, waste-based stabilizer (GSCFC) was proposed using five different industrial solid wastes: ground granulated blast-furnace slag (GGBS), steel slag (SS), coal fly ash (CFA), flue-gas desulfurization (FGD) gypsum, and carbide slag (CS). The mechanical and durability performance of GSCFC-stabilized soil were evaluated using unconfined compressive strength (UCS), California bearing ratio (CBR), and freeze–thaw and wet–dry cycles. The Rietveld method was employed to analyze the mineral phases in the GSCFC-stabilized soil. The optimal composition of the GSCFC stabilizer was determined as 15% SS, 12% GGBS, 16% FGD gypsum, 36% CS, and 12% CFA. The GSCFC-stabilized soil exhibited higher CBR values, with results of 31.38%, 77.13%, and 94.58% for 30, 50, and 98 blows, respectively, compared to 27.23%, 68.34%, and 85.03% for OPC. Additionally, GSCFC-stabilized soil demonstrated superior durability under dry–wet and freeze–thaw cycles, maintaining a 50% higher UCS (1.5 MPa) and a 58.6% lower expansion rate (3.16%) after 15 dry–wet cycles and achieving a BDR of 86.86% after 5 freeze–thaw cycles, compared to 65% for OPC. Rietveld analysis showed increased hydration products (ettringite by 2.63 times, C-S-H by 2.51 times), significantly enhancing soil strength. These findings highlight the potential of GSCFC-stabilized soil for durable road sub-base applications. This research provides theoretical and technical support for the development of sustainable, cost-effective, and eco-friendly soil stabilizers as alternatives to traditional cement-based stabilizers while also promoting the synergistic utilization of multiple solid wastes.

## 1. Introduction

Low liquid limit soil is classified as problematic soil in engineering, characterized by low strength, poor water stability, and high sensitivity to freeze–thaw cycles [1,2]. This type of soil is generally unsuitable for direct use in road construction material [3,4], leading to significant waste of valuable soil resources. To address this issue, the use of stabilizers has become a common and effective method for improving the engineering properties of problematic soils [5,6]. When the stabilizer is fully mixed with soil particles, a series of physical and chemical reactions will occur, improving the contact surfaces between soil particles, filling the voids between them, and enhancing the compressive strength of the soil. The most commonly used traditional soil stabilizers include ordinary Portland cement (OPC) and lime [7,8,9,10]. However, these stabilizers exhibit limitations. Lime-stabilized soil has low early compressive strength, slow strength development, a high drying shrinkage coefficient, and poor water stability, making it prone to dehydration, cracking, and softening when exposed to water [10,11]. Cement-stabilized soil exhibits rapid strength development in its early stages; however, its high shrinkage and thermal contraction coefficients render it susceptible to cracking. This limitation significantly decreases the pavement’s impermeability, erosion resistance, and overall structural integrity [12]. Moreover, cement shows limited effectiveness in stabilizing soils with high plasticity indices, such as expansive soils, clay, and organic soils [13]. Simply relying on only one traditional solidifying material, such as cement, often fails to achieve the expected reinforcement effect [14]. In seasonal areas, soil stabilized with traditional cement stabilizers exhibits significant degradation after freeze–thaw cycles [15,16], with subgrade strength reducing by approximately 50% [17]. Additionally, the production of cement and lime poses considerable environmental challenges [18,19]. Therefore, it is imperative to explore and develop innovative soil stabilizers as sustainable alternatives to conventional materials.

Statistics show that global solid waste production is expected to reach nearly 27 billion tons annually by 2050 [20]. Furthermore, many industrial wastes are not properly treated or utilized, often being stored or landfilled in open spaces, leading to waste of resources and land [21]. In recent years, the use of industrial soil waste as a soil stabilizer has attracted increasing attention. This approach not only enhances the utilization of solid waste resources but also helps reduce environmental pollution and carbon emissions [22,23,24,25]. This eco-friendly soil stabilizer utilizes industrial solid waste as its raw material, forming cementing substances through interactions among the components of solid waste. The aluminosilicate and calcium oxide minerals abundant in industrial solid waste undergo dissolution, diffusion, and hydration reactions in an alkaline solution environment, resulting in the formation of hydration products, e.g., calcium silicates hydrates (C-S-H) gel, calcium hydroxide, and ettringite [26,27]. These hydration products effectively bind soil particles, playing a crucial role in improving the compressive strength and mechanical performance of the stabilized soil [28]. Recent studies have shown that using multiple solid wastes simultaneously in soil stabilization can effectively combine the strengths of various waste materials, resulting in a synergistic stabilization effect and improved stabilization performance [29,30,31]. Additionally, soil stabilizers incorporating solid waste exhibit better freeze–thaw durability compared to traditional stabilizers [32].

The research gap is that while significant efforts have been made to stabilize problematic soils using industrial solid waste, there is still limited understanding of how different types of industrial waste can be synergistically combined to enhance both the mechanical properties and durability of stabilized low liquid limit soil, particularly in regions with repeated freeze–thaw cycles. The purpose of this article is to investigate the mechanical properties and durability performance of low liquid limit soil stabilized with an innovative industrial solid waste-based stabilizer, compared to traditional ordinary Portland cement stabilizers. In this study, ground granulated blast-furnace slag (GGBS), steel slag (SS), coal fly ash (CFA), flue-gas desulphurization gypsum (FGD) gypsum, and carbide slag (CS) were selected to prepare soil solidification agents (GSCFC) to enhance the mechanical properties and durability of liquid limit soil from Hebei Province, China. These industrial solid wastes are generally alkaline and exhibit pozzolanic activity, which can promote the progress of the hydration reaction [22,33]. Orthogonal experimental design was used to design the optimized ratio of raw materials. The low liquid limit soil used in this study is distributed in northern China, where repeated seasonal freeze–thaw cycles cause structural damage to stabilized soil. Therefore, the durability of the stabilized soil, such as dry–wet cycle and freeze–thaw cycle characteristics, was systematically studied in this research. The California bearing ratio (CBR) and unconfined compressive strength (UCS) are used to evaluate the bearing capacity and mechanical properties of stabilized soil. Its stabilization effects and performance were compared to those of low liquid limit soil stabilized with the same amount of ordinary Portland cement. The scanning electron microscopy analysis and Rietveld method were performed to study the microstructure and synergistic hydration mechanism. The innovation of this study lies in the development of a novel eco-friendly stabilizer using multiple types of industrial solid waste, achieving a synergistic stabilization effect that enhances both the mechanical properties and durability of low liquid limit soil. Furthermore, this study provides new insights into the hydration mechanism and microstructural evolution of stabilized soil, contributing to the development of sustainable alternatives to traditional cement-based stabilizers.

## 2. Materials and Methods

### 2.1. Materials

**Figure 1:**
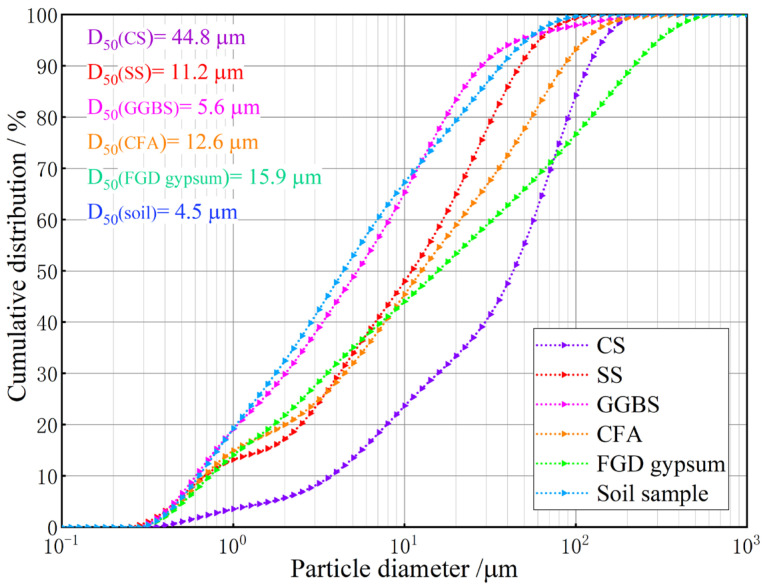
Particle size distribution curves of soil sample and industrial solid wastes.

**Figure 2:**
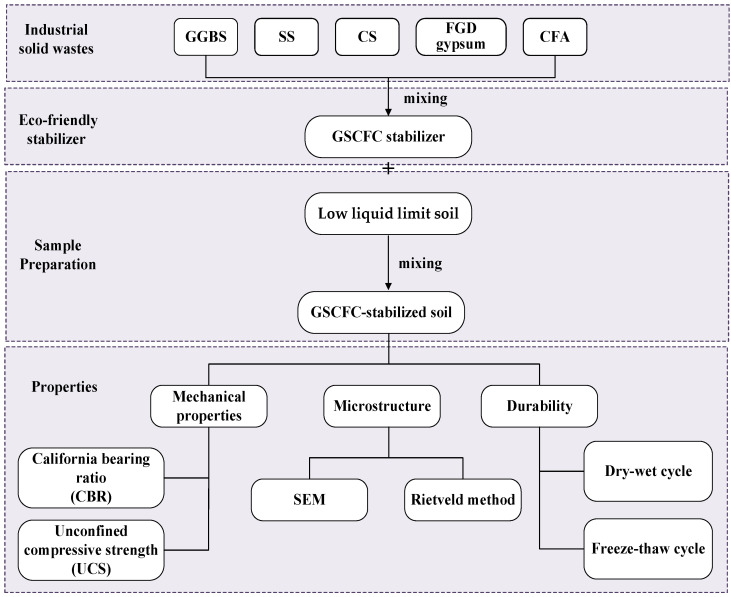
Flowchart of preparation and characterization of GSCFC-stabilized soil.

The low liquid limit soil used in this study was collected from Guanzhuang Township, Hebei Province, China, at a depth of 0.5 m below the cultivation layer. The particle size distribution curve of the soil sample is shown in Figure 1. The soil sample had a D_50_ of 4.5 μm, indicating a relatively fine particle size. The physical characteristics and chemical composition of the soil sample are presented in Table 1 and Table 2. The results showed that the liquid limit and plastic limit of the soil sample were 43.72% and 22.15%, respectively, with a plasticity index of 21.57. Therefore, the soil used in this study is classified as low liquid limit clay with a low plasticity index, exhibiting poor stability in the presence of water. These properties lead to compaction challenges and reduced strength, making it unsuitable for direct use in embankment construction. Table 2 shows that the main chemical components of the soil sample are SiO_2_, Al_2_O_3_, CaO, Fe_2_O_3_, and MgO. Figure 2 provides the XRD pattern of the soil sample, indicating that the primary mineral phases are quartz, plagioclase, chlorite, mica, and calcite.

The industrial solid wastes used in this study included GGBS, SS, CS, CFA, and FGD gypsum provided by China Railway Corp. (Cangzhou, China), Guohua Power Generation Co., Ltd. (Cangzhou, China) and Jinniu Chemical Co., Ltd. (Cangzhou, China). The grain size distribution curves of these solid wastes are shown in Figure 1. The D_50_ of GGBS was 5.6 μm, which closely matched the particle size of the soil sample. Their particle size distribution curves showed a high degree of similarity, suggesting that SS particles were as fine as soil samples. The D_50_ of SS, CS, CFA, and FGD gypsum were 11.2 μm, 44.8 μm, 12.6 μm, and 15.9 μm, respectively. Notably, CS had a broader particle size distribution range, with larger particles compared to the other solid wastes. The main chemical components of industrial solid waste are shown in Table 2. The dominant chemical components of GGBS were SiO_2_ (40.12%) and Al_2_O_3_ (31.42%). CFA, SS, and CS mainly consisted of CaO, with contents of 43.12%, 94.13%, and 39.92%, respectively; FGD gypsum was primarily composed of CaO (48.65%) and SO_3_ (43.51%). These components exhibited alkalinity and pozzolanic activity, enabling them to participate in hydration reactions [22].

### 2.2. Methods

#### 2.2.1. Sample Preparation

GSCFC stabilizer was prepared by mixing raw materials in a certain proportion, which is determined through an orthogonal experiment. A specific amount of soil stabilizer was added to the soil sample and mixed uniformly to obtain stabilized soil. The stabilized soil was placed in a sealed bag and stored for 24 h. Subsequently, the sample was placed in a cylindrical mold with a diameter and height of 50 mm × 50 mm and compacted using a TYA-2000 digital pressure testing machine (Lushida, Shaoxing, China). Afterward, the compacted sample was placed in a sealed bag and transferred to a standard environment with a relative humidity above 95% and a temperature maintained at 20 ± 2 °C for curing. After the curing period, the sample was tested for compressive strength and other properties. The flowchart of preparation and characterization of GSCFC-stabilized soil is shown in Figure 2.

#### 2.2.2. Orthogonal Experimental Design

Orthogonal experimental design is a method for studying experiments with multiple factors and multiple levels [34]. This method can improve experimental efficiency, optimize the content of each component, and determine the optimal component ratio for the preparation of the GSCFC stabilizer. A 4-factor, 4-level orthogonal test L16 (4^4^) was used to determine the optimal mass ratio of the raw materials, evaluating the effects of the dosages of SS (A), GGBS (B), and FGD gypsum (C), as well as the ratio of CS to CFA (D), on the unconfined compressive strength of the stabilized soil (Table 3 and Table 4). The influencing factors and levels are shown in Table 3, and the orthogonal test design is presented in Table 4.

Range analysis was used to evaluate the results of the orthogonal experiment and to determine the sensitivity of each factor on unconfined compressive strength. The average value *ki* and the influence degree *R* are calculated by Equations (1) and (2).

(1) $$k_{i,j}=\bar{P\left(k_{i,j}\right)}\left(i=A,B,C,D,j=1,2,3,4\right)$$

(2) $$R_{i}=\underset{i=A,B,C,D}{\max}\left(k_{i,1},k_{i,2},k_{i,3},k_{i,4}\right)-\underset{i=A,B,C,D}{\min}\left(k_{i,1},k_{i,2},k_{i,3},k_{i,4}\right)$$

where *k_i_*_,*j*_ is the average value of test results at level *j* of factor *i*; *P*(*k_i_*_,*j*_) is the result corresponding to the level *j* of factor *I*; *R_i_* is the range of *k_i_*_,*j*_ values corresponding to each level of factor *i*.

#### 2.2.3. Compressive Strength Test

The unconfined compressive strengths (UCS) were tested according to Chinese standard GB/T17671 [35] using an electro-hydraulic universal testing machine (CTS-E200) (Quanlitet, Jinan, China) with a loading rate of 1 mm/min. The measurements were performed on at least three representative samples to obtain an average value.

#### 2.2.4. California Bearing Ratio

The California bearing ratio (CBR) is an important indicator for evaluating the load-bearing capacity of subgrade materials. It reflects the material’s ability to resist deformation under localized load penetration; a higher CBR indicates a stronger subgrade [36]. The stabilized soil, after being sealed for 24 h, was subjected to a compaction test with compaction counts of 30, 50, and 98 blows. Each compaction count was tested in triplicate, and the CBR value of the stabilized soil was measured according to JTG 3430-2020 [37]. The compacted samples were placed in a water tank and soaked for 4 days, ensuring the water level was above the top of the sample containers. After soaking, the specimens were removed, wiped dry, and placed on the sample platform to ensure full contact between the penetration rod and the top surface of the specimen. The loading rate of the penetration rod was maintained at 1 mm/min, and the pressure was recorded at different penetration levels. The CBR at penetration depths of 2.5 mm and 5.0 mm was calculated using Equations (3) and (4), and the expansion rate of the stabilized soil was calculated using Equation (5).

When the penetration is 2.5 mm:

(3) $$\mathrm{CBR}=\frac{P}{7000}\times 100\%$$

When the penetration is 5 mm:

(4) $$\mathrm{CBR}=\frac{P}{10500}\times 100\%$$

where *CBR* is the bearing ratio of the specimen, accurate to 0.1%; *P* is the unit pressure during penetration (kPa).

The expansion rate is as follows:

(5) $$A=\frac{H_{1}-H_{0}}{H_{0}}\times 100\%$$

where *A* represents the expansion rate of the specimen after soaking, accurate to 0.1%; *H*_1_ is the height of the specimen at the end of soaking (mm); *H*_0_ is the initial height of the specimen (mm).

#### 2.2.5. Drying and Wetting Cycle

Drying and wetting cycle resistance indicates the stability of stabilized soil under alternating dry and wet conditions. After a 28-day curing period, the specimen’s compressive strength is measured to serve as the initial strength for the drying and wetting cycle test. The sample is immersed in water for 12 h for humidification, then placed indoors for 12 h for drying. One humidification-drying process is considered one dry–wet cycle. The unconfined compressive strength of the sample is tested following each dry–wet cycle.

#### 2.2.6. Freeze–Thaw Cycle 

Frost resistance is one of the key indicators for evaluating the durability of pavement materials. It refers to the ratio of unconfined compressive strength (UCS) of stabilized soil cured for 28 days after undergoing several freeze–thaw cycles to the UCS before the freeze–thaw cycles. The freeze–thaw cycle test involves placing samples cured for 28 days in a freezer at −20 °C for 16 h, followed by immersion in a constant-temperature water bath at 20 °C for 8 h. One freeze–thaw cycle consists of a complete freezing and thawing process. After each cycle, the compressive strength of the stabilized soil is measured and recorded. Additionally, the mass of the specimens is measured before and after the cycles, and the test is terminated if the mass loss exceeds 5%. The calculation of freeze–thaw resistance (*BDR*) is as follows:

(6) $$\mathrm{BDR}=\frac{R_{DC}}{R_{C}}\times 100\%$$

where *BDR* represents the unconfined compressive strength loss of the specimen after *n* freeze–thaw cycles, accurate to 0.1%; *R_DC_* is the unconfined compressive strength of the specimen (MPa) after *n* freeze–thaw cycles; *R_C_* is the unconfined compressive strength of the specimen under standard curing conditions.

#### 2.2.7. Rietveld Method

The Rietveld method for structural refinement is a technique for analyzing crystal structures and phase composition using X-ray diffraction (XRD) data, proposed by Dutch crystallographer Hugo M. Rietveld [38]. The principle of the Rietveld method is to fit the calculated XRD pattern to the measured pattern using the least squares method, aiming to minimize the difference between them. The result of the Rietveld refinement is evaluated by the weighted profile factor *R_wp_* and the expected factor *R_exp_*:

(7) $$R_{\mathrm{wp}}=\sqrt{\frac{\sum w_{i}{\left(y_{\mathrm{io}}-y_{\mathrm{ic}}\right)}^{2}}{\sum w_{i}{{\;y}_{\mathrm{io}}}^{2}}}$$

(8) $$R_{\exp}=\sqrt{\frac{\sum \left(N-P\right)}{\sum w_{i}{{\;y}_{\mathrm{io}}}^{2}}}$$

where *y_io_* and *y_ic_* are the observed and calculated intensities at point *i*, *w_i_* is the weight assigned to each intensity, *N* is the number of data points in the diffraction pattern, and *P* is the number of adjustable parameters in the fitting. It is generally considered that the smaller the *R*-value, the better the pattern fit and the more reliable the qualitative and quantitative data obtained. The BGMN program (version 5.1.8) was used for Rietveld analysis in this study.

#### 2.2.8. Testing Methods

The microstructure of samples was investigated by the scanning electron microscopy (SEM) analysis, using the JSM-6701F cold field emission scanning electron microscope from Shimadzu, Kyoto, Japan, and the Thermo NS7 energy spectrometer (Thermo, Waltham, MA, USA). The X-ray powder diffraction (XRD) analysis was performed on a Bruker Advance D8 X-ray diffractometer (Bruker, Billerica, MA, USA), utilizing Cu Kα radiation (λ = 0.1542 nm) with an operating voltage of 20 kV and a current of 200 mA. The scanning angle range was 5° to 80°, with a scanning speed of 5°/min.

## 3. Results and Discussion

### 3.1. Orthogonal Experimental Results Analysis

The results of the orthogonal experiment and range analysis are shown in Table 4 and Table 5, respectively. The influence of each factor on the 7-day unconfined compressive strength (7 d UCS) of the stabilized soil was, in order, A (SS) > B (GGBS) > D (CS:CFA) > C (FGD gypsum) (Table 5). Among all factors, SS had a greater impact on 7 d UCS of the stabilized soil, while FGD gypsum had a lesser effect. The 7 d UCS of the stabilized soil showed a decreasing trend with increasing SS content, whereas it increased with higher GGBS content, indicating that SS content has a negative impact on compressive strength, while slag has a positive impact. Additionally, as the amounts of FGD gypsum and CS increased, the strength initially increased and then decreased. Based on the results of range analysis, the optimal ratio for the GSCFC stabilizer was determined to be A_1_B_4_C_4_D_3_, which corresponds to contents of 15% SS, 12% GGBS, 16% FGD gypsum, 36% CS, and 12% CFA, with a carbide slag to fly ash ratio of 3:1.

### 3.2. Unconfined Compressive Strength Test

**Figure 3:**
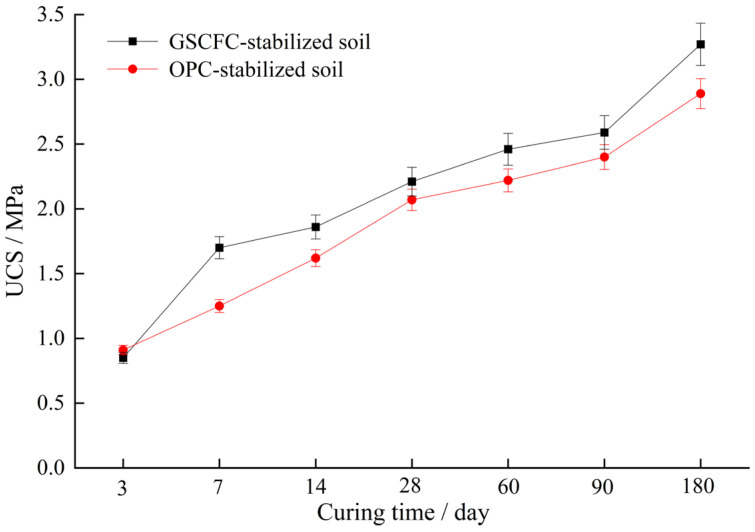
UCS of GSCFC-stabilized and OPC-stabilized soil.

The unconfined compressive strength (UCS) of stabilized soil with both the GSCFC stabilizer and ordinary Portland cement (OPC) was measured and compared to evaluate the stabilization effects. The addition of both two stabilizers was fixed at 12% of the total soil sample mass. The UCS of the two stabilized soil at curing ages of 3, 7, 14, 28, 60, 90, and 180 days are presented in Figure 3. As the curing period increased from 7 days to 180 days, the UCS of OPC-stabilized soil increased from 0.91 MPa to 2.84 MPa, while the UCS of GSCFC-stabilized soil increased from 0.80 MPa to 3.31 MPa. This indicates that the strength of the stabilized soil is positively correlated with the curing age [26,39]. During the first 3 days of curing, the UCS of OPC-stabilized soil was slightly higher than that of GSCFC-stabilized soil due to the relatively rapid early hydration rate of cement, which accelerated early strength development [29,40]. However, by the 7 days of curing, the UCS of GSCFC-stabilized soil was higher than OPC-stabilized soil and continued to increase with further curing. When the curing age was extended to 180 days, the UCS of the GSCFC-stabilized soil was 16.55% higher than that of the OPC-stabilized soil. It is evident that the solidification effect of the GSCFC stabilizer is better than that of the OPC stabilizer, which is consistent with the results of previous studies [23,41].

### 3.3. California Bearing Ratio Analysis

**Figure 4:**
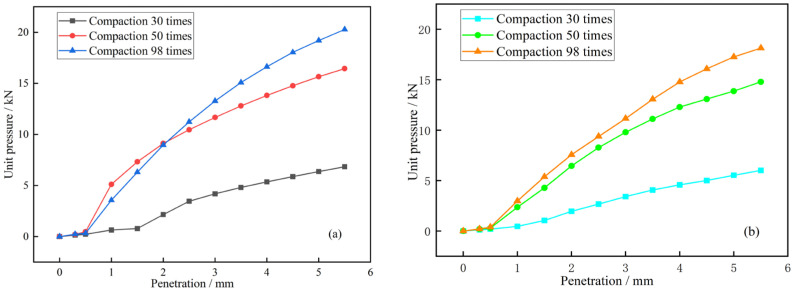
Relationship between unit pressure and penetration: (**a**) GSCFC-stabilized soil; (**b**) OPC-stabilized soil.

It is generally considered that the number of blows in the bearing ratio test corresponds to the degree of compaction: 30 blows correspond to 92% compaction, 50 blows to 95% compaction, and 98 blows to 100% compaction. For each compaction blow count, three parallel specimens were prepared, and the arithmetic mean was taken as the test result. The larger CBR value at penetration depths of 2.5 mm and 5 mm was used as the bearing ratio of the material. Figure 4a,b shows the relationship between unit pressure and penetration for soil stabilized with GSCFC stabilizer and OPC under different numbers of blows, respectively. Table 6 presents the expansion rate and bearing ratio of GSCFC-stabilized soil and OPC-stabilized soil.

It could be seen from Figure 4 that at the same penetration level, the load pressure for both GSCFC-stabilized and OPC-stabilized soil increased with the number of blows, and the load pressure for the GSCFC-stabilized soil was greater than that of the OPC-stabilized soil. According to Table 6, the CBR values for GSCFC-stabilized soil are 31.38%, 77.13%, and 94.58% for 30, 50, and 98 blows, respectively. In comparison, the OPC-stabilized soil shows CBR values of 27.23%, 68.34%, and 85.03% for the same respective blow counts. The CBR values of both types of stabilized soil were significantly higher than the 8% required by “Technical Specifications for Highway Subgrade Construction” (JTG/T3610-2019) in China [42]. At all three different compaction levels, the CBR of the GSCFC-stabilized soil is higher than that of the OPC-stabilized soil. As the number of compaction blows increased, the value of CBR rose accordingly. The greater the number of blows, the larger the difference in CBR between the two types of stabilized soil. Additionally, the expansion rate increased with the number of compaction blows [43], and the expansion rate of the GSCFC-stabilized soil was lower than that of OPC-stabilized soil. These results suggest that, compared to OPC-stabilized soil, the GSCFC-stabilized soil has greater resistance to damage and better water stability, making it more suitable as a sub-base material for roads.

### 3.4. Dry–Wet Cycle Analysis

**Figure 5:**
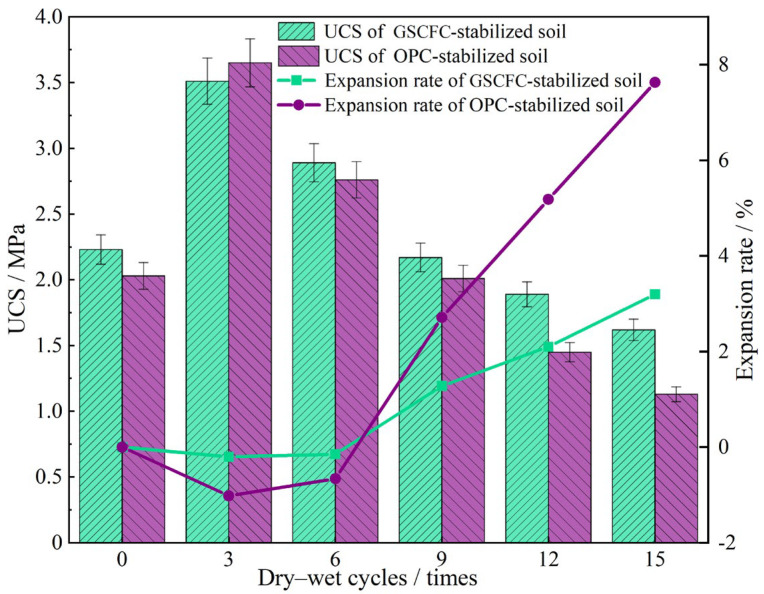
Variation diagram of compressive strength and expansion rate under different dry–wet cycles.

Dry–wet cycle tests were conducted on GSCFC-based and OPC-based stabilized soils with a curing age of 28 days. The number of cycles was set at 3, 6, 9, 12, and 15, respectively. Following each specified number of cycles, the UCS and expansion rate of the specimens were measured, and their variation trends are illustrated in Figure 5. During 1 to 6 dry–wet cycles, the expansion rates of both types of stabilized soil were negative, indicating varying degrees of shrinkage. The UCS of GSCFC-stabilized soil also increased and was higher than that of the OPC-stabilized soil, which had not undergone dry–wet cycling. The main reason for this phenomenon was that, during the initial dry–wet cycles, the stabilized soil specimens primarily experienced moisture evaporation and loss, leading to shrinkage, which made them harder and more susceptible to erosion. After more than six dry–wet cycles, the internal spatial structure of the stabilized soil was damaged, and the soil particles began to absorb water and expand. With the increasing number of cycles, the expansion rate gradually increased, the UCS gradually decreased, and varying degrees of spalling began to appear around the specimens [39].

By comparing the two types of stabilized soil, it can be observed that after three dry–wet cycles, the UCS of the OPC-stabilized soil was slightly higher than that of the GSCFC-stabilized soil, with a wider range of shrinkage and expansion. This difference can be attributed to the greater tendency of conventional cementitious material to lose moisture and shrink. After 6 dry–wet cycles, the UCS of the OPC-stabilized soil decreased rapidly, dropping to just 1.0 MPa after 15 cycles. In contrast, the GSCFC-stabilized soil demonstrated greater stability, maintaining a compressive strength of 1.5 MPa after 15 cycles, which was 50% higher than that of the cement-stabilized soil. After 15 dry–wet cycles, the expansion rate of the GSCFC-stabilized soil was 3.16%, which was 58.6% lower than that of the OPC-stabilized group. This indicated that the GSCFC-stabilized soil had better resistance to the drying and wetting cycle and superior durability compared to the cement-stabilized soil.

**Figure 6:**
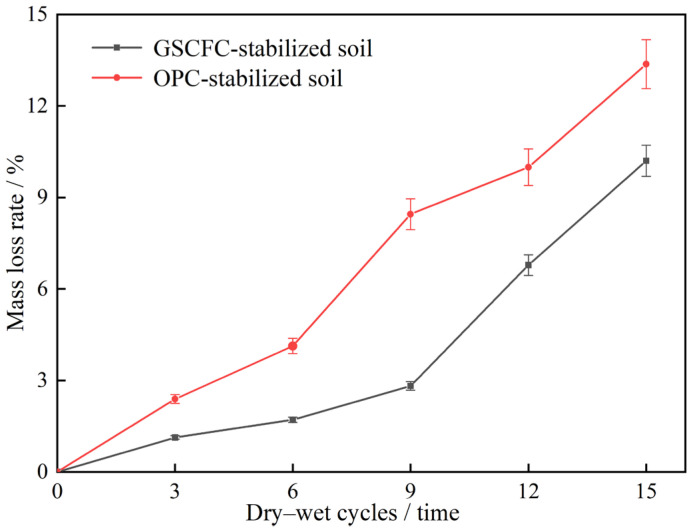
Effect of dry–wet cycle on mass of two kinds of solidified soil.

Figure 6 shows the mass loss rate results for two types of stabilized soil. The results showed that the mass loss rate for both types of stabilized soil gradually increased with the number of dry–wet cycles [39]. Throughout the 15 cycles, the mass loss rate of the OPC-stabilized soil was consistently higher than that of GSCFC-stabilized soil. Before nine dry–wet cycles, the growth rate of mass loss for GSCFC-stabilized soil was relatively low, with approximately 50% less mass loss compared to the OPC-stabilized soil. The mass loss rate of the OPC-stabilized soil increased rapidly after six dry–wet cycles. This was due to the gradual deterioration at the edges and corners of the OPC-stabilized specimens, resulting in substantial mass loss. For GSCFC-stabilized soil, similar phenomena began to appear after nine dry–wet cycles. This further indicated that the stability of the GSCFC-stabilized soil was higher than that of the OPC-stabilized soil.

### 3.5. Freeze–Thaw Cycle Analysis

**Figure 7:**
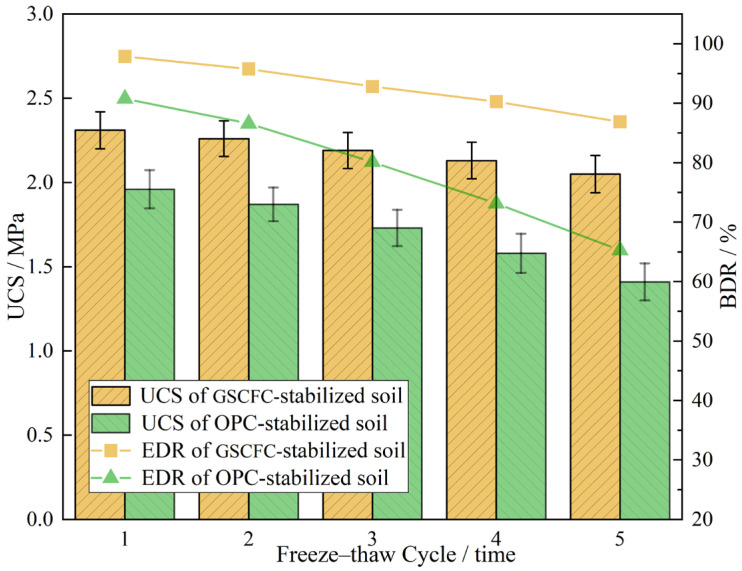
Variation of compressive strength with the number of freeze–thaw cycles.

Hebei Province, located in northern China, where winter temperatures can drop to below minus ten degrees Celsius. Therefore, the frost resistance of pavement base materials should be considered. The freeze–thaw cycle tests were conducted on GSCFC-stabilized and OPC-stabilized soil cured for 28 days under standard conditions, and the experimental results are shown in Figure 7.

**Figure 8:**
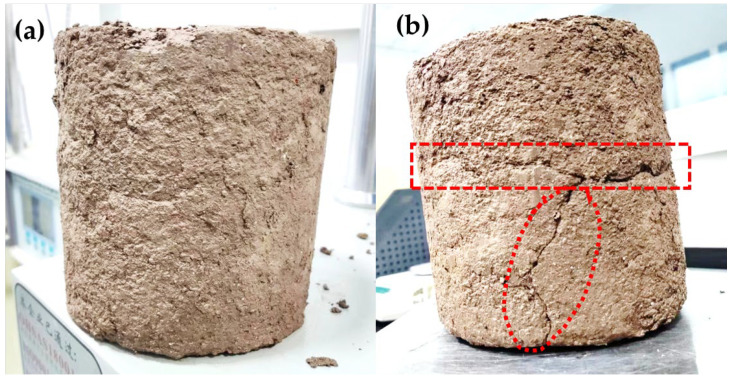
Stabilized soil after 5 freeze–thaw cycles: (**a**) GSCFC-stabilized soil; (**b**) OPC-stabilized soil.

The results indicated that with the increase in the number of freeze–thaw cycles, the UCS values, and the freeze–thaw resistance indicator (BDR) of GSCFC-stabilized and OPC-stabilized soil gradually decreased. This is because the decrease in compressive strength of samples after freeze–thaw cycles is due to the redistribution and freezing of water within the sample, which damages its internal structure and reduces stability. Repeated freeze–thaw cycles loosen the soil structure, increase porosity, weaken the cohesion between soil particles, and ultimately lead to a decrease in UCS and BDR [44,45]. The values for GSCFC-stabilized soil are higher than those for OPC-stabilized soil. After five freeze–thaw cycles, the compressive strength of the stabilizer-stabilized soil showed a minimal reduction, with a freeze–thaw resistance indicator (BDR) of 86.86%, meeting the requirement of no less than 80%, as specified in the Technical Standard for Soil Stabilizer Application. Figure 8 shows that after five freeze–thaw cycles, the GSCFC-stabilized soil exhibited almost no visible changes in appearance. In contrast, the OPC-stabilized soil exhibited cracking, accompanied by a significant reduction in compressive strength, with a BDR value of approximately 65%. This indicates that the GSCFC-stabilized soil has superior frost resistance, which is attributed to the synergistic effect among the different solid waste materials [44].

### 3.6. Rietveld Refinement Analysis

**Figure 9:**
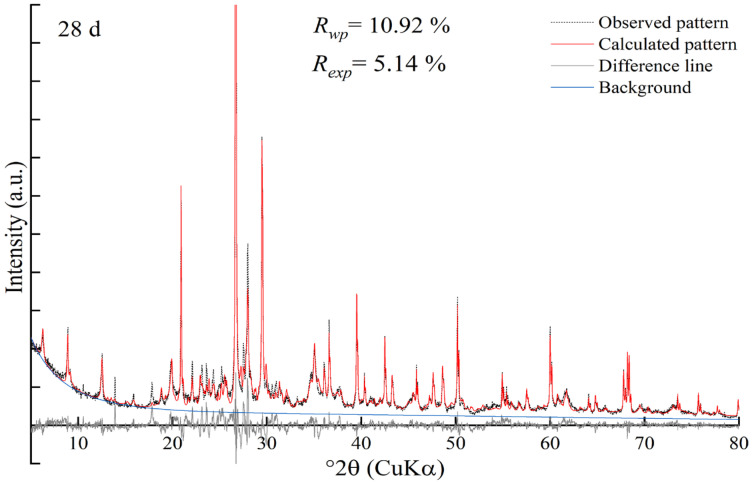
Rietveld refinements of GSCFC-stabilized soil at curing age 28 days.

The Rietveld method was employed to investigate the mineral phases of stabilized soil at different curing ages, and its accuracy was evaluated by the weighted profile factor *R*_wp_ and the expected factor *R*_exp_. Figure 9 presents the Rietveld analysis results for the GSCFC-stabilized soil cured for 28 days. It can be seen from Figure 9 that the calculated patterns showed a high degree of agreement with the measured patterns, indicating high credibility. The Rietveld results for the GSCFC-stabilized soil after 3, 7, 28, and 90 days of curing are displayed in Table 7 and Figure 9. The *R*_wp_ values ranged from 9.78% to 11.88%, and the *R*_exp_ values ranged from 4.86% to 5.17%.

**Figure 10:**
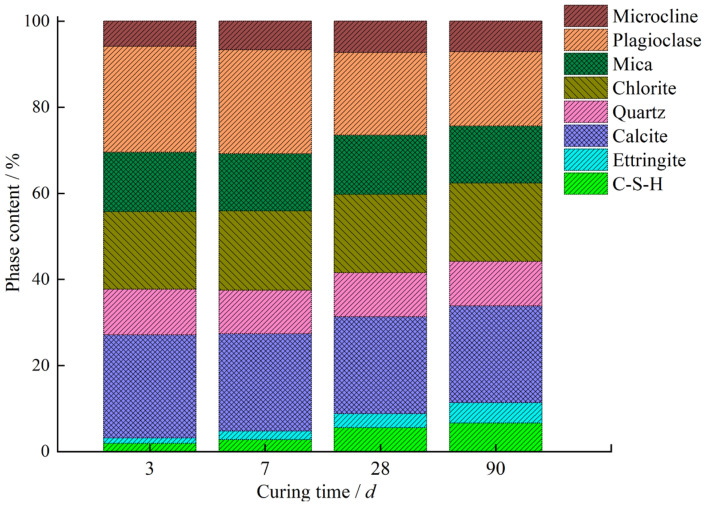
Variation of mineral phase content in GSCFC-stabilized soil at different curing ages.

The results showed that the main mineral phases in the stabilized soil were quartz (SiO_2_), chlorite ((Fe, Mg)_5_Al(Si_3_Al)O_10_(OH)_8_), mica (KAl_2_(AlSi_3_O_10_)(OH)_2_), plagioclase ((Na, Ca)(Si, Al)_4_O_8_) and calcite (CaCO_3_). The primary hydration products were ettringite (3CaO·Al_2_O_3_·3CaSO_4_·32H_2_O) and calcium silicate hydrate (C-S-H, CaO·SiO_2_·*n*H_2_O); their content increased with the extension of curing time (Table 7, Figure 10). When the raw materials were mixed with water, a large number of ions were released, such as OH^−^, Ca^2+^, SO_4_^2−^, and Al^3+^. These ions reacted with each other, promoting the progression of the hydration reaction and resulting in the formation of hydration products like C-S-H and ettringite [29,31,39,46].

(9) $${\mathrm{SiO}}_{2}+H_{2}O+{\mathrm{OH}}^{-}\to {\;\left[H_{3}{\mathrm{SiO}}_{4}\right]}^{-}+{\mathrm{Ca}}^{2+}\to C-S-H$$

(10) $$2[\mathrm{Al}(\mathrm{OH}{)}_{6}{]}^{3-}+{6Ca}^{2+}+{3SO}_{4}^{2-}+26H_{2}O\to 3CaO\cdot {Al}_{2}O_{3}\cdot 3{CaSO}_{4}\cdot 32H_{2}O$$

The ettringite content increased from 1.31% (3 days) to 4.76% (90 days), an increase of approximately 2.63 times; the C-S-H content increased from 1.89% (3 days) to 6.63% (90 days), an increase of approximately 2.51 times (Table 7). This indicated that as the hydration reaction progressed, the amount of hydration products C-S-H and ettringite continuously increased, making the internal structure of the stabilized soil denser and enhancing the UCS, which corresponded to the results of the UCS analysis (Figure 3). No diffraction peaks of gypsum, dicalcium silicate, or tricalcium silicate were observed among the hydration products, suggesting that they participated in the hydration reaction at an early stage and were already consumed. The content of minerals such as quartz, calcite, chlorite, and mica remained essentially unchanged before and after curing (Figure 10), indicating they did not participate in the hydration reaction.

### 3.7. SEM Analysis

**Figure 11:**
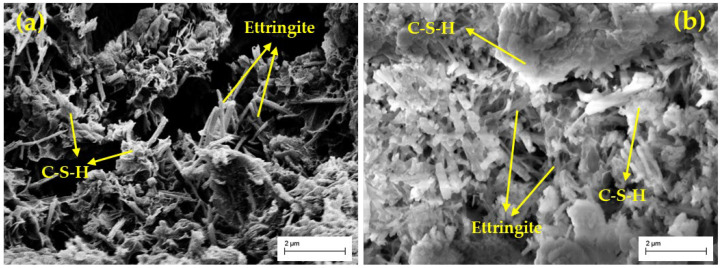
SEM images of GSCFC-stabilized soil at different curing ages: (**a**) 7 days; (**b**) 90 days.

Figure 11 shows the microstructure of the stabilized soil at different curing ages. In Figure 11a, needle-like ettringite was observed in the stabilized soil at the curing age of 7 days, and C-S-H gel was also formed. As the curing age increased, more C-S-H gel was observed in the stabilized soil at 90 days (Figure 11b), and the ettringite gradually transformed into relatively thicker rod-like formations distributed within the soil matrix. The C-S-H gel and ettringite interconnected, forming a stable three-dimensional network structure that effectively filled the pore spaces within the stabilized soil, making the soil structure denser and enhancing the compressive strength [29,31]. These conclusions are consistent with the results of the UCS and Rietveld analyses

## 4. Conclusions

In this study, an eco-friendly soil stabilizer, GSCFC, which is composed of five industrial solid wastes, GGBS, SS, CFA, FGD gypsum, and CS as raw materials, was developed for stabilizing low liquid limit soil. The mechanical properties and durability of GSCFC-stabilized soil were investigated and compared with those of OPC-stabilized soil. The following conclusions have been drawn:

(1) The optimal composition for GSCFC stabilizer determined by orthogonal experimental design was 15% SS, 12% GGBS, 16% FGD gypsum, 36% CS, and 12% CFA. In the first 3 days of curing, OPC-stabilized soil showed slightly higher UCS due to its faster early hydration. By day 7, GSCFC-stabilized soil surpassed the OPC group in UCS and continued to strengthen with further curing;
(2) The GSCFC-stabilized soil showed higher CBR values than OPC-stabilized soil at all compaction levels, with values of 31.38%, 77.13%, and 94.58% for 30, 50, and 98 blows, respectively, compared to 27.23%, 68.34%, and 85.03% for OPC group. Both exceeded the 8% minimum required by China’s highway subgrade standards, and the GSCFC-stabilized soil exhibited better water stability and resistance to damage, making it more suitable for use as road sub-base material;
(3) The GSCFC-stabilized soil demonstrated significantly better durability under dry–wet cycles, with a 50% higher UCS (1.5 MPa) and a 58.6% lower expansion rate (3.16%) compared to OPC-stabilized soil after 15 cycles. Moreover, the mass loss rate of GSCFC-stabilized soil was consistently lower than that of OPC-stabilized soil, with approximately 50% less mass loss before nine cycles. After five freeze–thaw cycles, the GSCFC-stabilized soil demonstrated superior frost resistance, with a compressive strength reduction of only 13.14% and a BDR of 86.86%, exceeding the requirement of 80%. In comparison, OPC-stabilized soil exhibited visible cracking and a significant strength reduction, with a BDR of approximately 65%. This indicated that GSCFC-stabilized soil showed higher stability and better resistance to shrinkage and structural degradation;
(4) The Rietveld results indicated that the primary hydration products were ettringite and C-S-H. As the curing period extended from 3 to 90 days, the ettringite content increased by approximately 2.63 times (from 1.31% to 4.76%), and the C-S-H content increased by about 2.51 times (from 1.89% to 6.63%), significantly enhancing the compressive strength of GSCFC-stabilized soil.

The results of this study have significant importance for the road maintenance industry, highlighting the potential of GSCFC-stabilized soil for subbase material in road construction, particularly in regions with harsh climatic conditions, such as freeze–thaw cycles. Compared to traditional cement-based soil stabilizers, the GSCFC stabilizer exhibits superior mechanical performance, enhanced durability, and environmental friendliness. Moreover, this kind of stabilizer promotes the utilization of industrial solid waste, providing a cost-effective and eco-friendly solution for road-based applications. 

## Figures and Tables

**Table 1:** Physical and mechanical properties of soil sample.

Natural Water Content/%	Optimum Water Content/%	Maximum Dry Density/g·cm^−3^	Plastic Limit/%	Liquid Limit/%	Plasticity Index
9.36	15.81	1.82	22.15	43.72	21.57

**Table 2:** Chemical composition of soil sample and industrial solid wastes (wt.%).

	Soil Sample	GGBS	CFA	CS	SS	FGD Gypsum
SiO_2_	56.63	40.12	27.56	2.13	16.02	2.93
Al_2_O_3_	16.75	31.42	15.83	1.95	2.98	1.04
CaO	9.66	14.85	43.12	94.13	39.92	48.65
SO_3_	0.10	1.15	2.67	0.27	0.23	43.51
Fe_2_O_3_	7.24	6.99	1.60	0.30	26.26	0.98
MgO	3.78	0.95	7.01	-	7.84	1.42
K_2_O	3.59	1.22	0.36	-	0.02	0.21
Na_2_O	0.92	0.89	0.35	0.49	0.37	0.43
P_2_O_5_	0.27	0.62	0.03	0.01	2.52	0.02
Others	1.06	1.79	1.48	0.72	3.83	0.80

**Table 3:** Factors and levels of orthogonal test.

Levels	Proportions/%			
	A (SS)	B (GGBS)	C (FGD Gypsum)	D (CS:CFA)
1	15	6	10	1:1
2	20	8	12	2:1
3	25	10	14	3:1
4	30	12	16	4:1

**Table 4:** Schemes and results of orthogonal experimental design.

Number	A	B	C	D	7 d UCS/MPa
1	1	1	1	1	1.29
2	1	2	2	2	1.54
3	1	3	3	3	1.76
4	1	4	4	4	1.65
5	2	1	2	3	1.43
6	2	2	1	4	1.46
7	2	3	4	1	1.49
8	2	4	3	2	1.73
9	3	1	3	4	1.25
10	3	2	4	3	1.44
11	3	3	1	2	1.45
12	3	4	2	1	1.41
13	4	1	4	2	1.13
14	4	2	3	1	1.22
15	4	3	2	4	1.28
16	4	4	1	3	1.40

**Table 5:** Analysis of range of orthogonal experiment.

Levels	7 d UCS/MPa			
	A (SS)	B (GGBS)	C (FGD Gypsum)	D (CS:CFA)
*k_i_*_,1_	2.15	1.67	1.83	1.78
*k_i_*_,2_	2.01	1.90	1.86	1.93
*k_i_*_,3_	1.82	1.98	1.90	2.03
*k_i_*_,4_	1.72	2.05	1.97	1.86
*R_i_*	0.47	0.39	0.12	0.28
Ranking	A > B > D > C			
Optimum theme	A_1_B_4_C_4_D_3_			

**Table 6:** Expansion rate and CBR value of solidified soil with curing agent under different compaction times.

		Blows/Time		
		30	50	98
GSCFC-stabilized soil	Expansion rate/%	1.26	1.01	0.96
	CBR/%	31.38	77.13	94.58
OPC-stabilized soil	Expansion rate/%	1.4	1.2	1.06
	CBR/%	27.23	68.34	85.03

**Table 7:** The Rietveld refinement results of GSCFC-stabilized soil at different curing ages.

Minerals	Content/wt. %			
	3 d	7 d	28 d	90 d
	*R*_wp_ = 10.62% *R*_exp_ = 4.94%	*R*_wp_ = 9.78% *R*_exp_ = 4.86%	*R*_wp_ = 10.92% *R*_exp_ = 5.17%	*R*_wp_ = 11.88% *R*_exp_ = 5.14%
C-S-H	1.89	2.82	5.57	6.63
Ettringite	1.31	1.93	3.24	4.76
Plagioclase	24.62	24.19	19.21	17.26
Calcite	23.96	22.65	22.52	22.47
Chlorite	18.04	18.43	18.15	18.21
Mica	13.81	13.27	13.78	13.26
Quartz	10.57	10.12	10.28	10.32
Microcline	5.81	6.58	7.25	7.10
sum	100.01	99.99	100.00	100.01

## Data Availability

The original contributions presented in this study are included in the article. Further inquiries can be directed to the corresponding author.
